# ESBL-Producing and Macrolide-Resistant *Shigella sonnei* Infections among Men Who Have Sex with Men, England, 2015

**DOI:** 10.3201/eid2211.160653

**Published:** 2016-11

**Authors:** Piers Mook, Jacquelyn McCormick, Manpreet Bains, Lauren A. Cowley, Marie A. Chattaway, Claire Jenkins, Amy Mikhail, Gwenda Hughes, Richard Elson, Martin Day, Rohini Manuel, Jayshree Dave, Nigel Field, Gauri Godbole, Timothy Dallman, Paul Crook

**Affiliations:** Public Health England, London, UK (P. Mook, J. McCormick, M. Bains, L.A. Cowley, M.A. Chattaway, C. Jenkins, A. Mikhail, G. Hughes, R. Elson, M. Day, N. Field, G. Godbole, T. Dallman, P. Crook);; Barts Health National Health Service Trust, London (R. Manuel, J. Dave);; University College London, London (N. Field)

**Keywords:** Shigella, Shigella sonnei, bacteria, bacterial infections, sexually transmitted infections, STIs, men who have sex with men, MSM, outbreaks, antimicrobial resistance, England, ESBL producers, extended-spectrum beta-lactamase, ESBL

## Abstract

In England in 2015, *Shigella sonnei* isolates from men who have sex with men produced extended-spectrum β-lactamases and exhibited macrolide resistance. Whole-genome sequencing showed a close relationship among the isolates, which harbored a plasmid that was previously identified in a shigellosis outbreak among this population but has acquired a mobile element.

Historically, shigellosis is an infection associated with travel to countries in which this infection is endemic. In England in 2009, *Shigella flexneri* 3a infections dramatically increased among men who have sex with men (MSM) and were thought to be associated with sexual transmission (*1*). Since 2011, an emerging epidemic of *S. sonnei* infections in England among men has occurred (*2*). We describe an investigation of a cluster of multidrug-resistant *S. sonnei* infections among MSM in England during 2015.

## The Study

In November 2015, Public Health England (PHE) identified a cluster of *S. sonnei* isolates by analyzing whole-genome sequencing data from specimens collected during September and October from 4 adult men residing in London and having no known travel history. Clinical reporting data indicated that 2 of the men were MSM. The isolates were later found to exhibit high levels of antimicrobial drug resistance to amoxicillin, ceftriaxone, tetracycline, sulfonamides, trimethoprim, and azithromycin and to produce extended-spectrum β-lactamase (ESBL) (Table 1). The isolates were sensitive to quinolones. PHE convened an outbreak control team to identify additional cases, characterize cases, capture exposure information, rule out a point source for the outbreak, and provide evidence for additional targeted public health efforts.

**Table 1:** Results of the phenotypic antimicrobial drug resistance profile of 4 isolates of *Shigella sonnei*, England, 2015*

Isolate ID	MIC for antimicrobial drugs tested, mg/mL†																	
	AMP	AMC	CTX	CRO	CAZ	CFP	GEN	TOB	TMP	SEP	ATM	CIP	COL	ERT	MER	FOS	TEM	AZT
182834	>1,052	1	>32	>32	2	0.38	0.5	0.38	>32	64	0.38	<0.032	0.19	0.008	0.06	1	<1	>256
184494	>1,052	1	>32	>32	2	0.5	0.38	0.38	>32	128	0.5	<0.032	0.125	0.008	0.06	1.5	<1	>256
183660	>1,052	1	>32	>32	1	0.38	0.38	0.38	>32	128	0.38	<0.032	0.125	0.008	0.06	1	<1	>256
164679	>1,052	1	>32	>32	1	0.38	0.38	0.38	>32	64	0.38	<0.032	0.19	0.008	0.06	1	<1	>256

*TEM was included in the panel to aid detection of OXA-48–like carbapenemases. AMC, amoxicillin/clavulanate; AMP, ampicillin; ATM, aztreonam; AZT, azithromycin; CAZ, ceftazidime; CFP, cefepime; CIP, ciprofloxacin; COL, colistin; CRO, ceftriaxone; CTX, cefotaxime; ERT, ertapenem; FOS, fosfomycin; GEN, gentamicin; ID, identification; MER, meropenem; SEP, trimethoprim/sulfamethoxazole; TEM, temocillin; TMP, trimethoprim; TOB, tobramycin.  †Phenotypic antimicrobial drug susceptibility testing was performed by using Etest (bioMérieux, Marcy l’Etoile, France) and interpreted by using breakpoints for *Enterobacteriaceae* established by the European Committee on Antimicrobial Susceptibility Testing (http://www.eucast.org/). Production of extended-spectrum β-lactamase was confirmed by using CTX + clavulunic acid and CAZ + clavalunic acid in combination (bioMérieux).

Since August 2015, PHE has conducted whole-genome sequencing of ≈70% of *S. sonnei* isolates (those voluntarily referred from hospital laboratories) (Technical Appendix). Single nucleotide polymorphism (SNP) clustering is performed, as previously described (*3*). Readings (in FASTQ format) from all sequences in this study can be found at the PHE Pathogens BioProject (National Center for Biotechnology Information project no. PRJNA315192).

**Figure 1:**
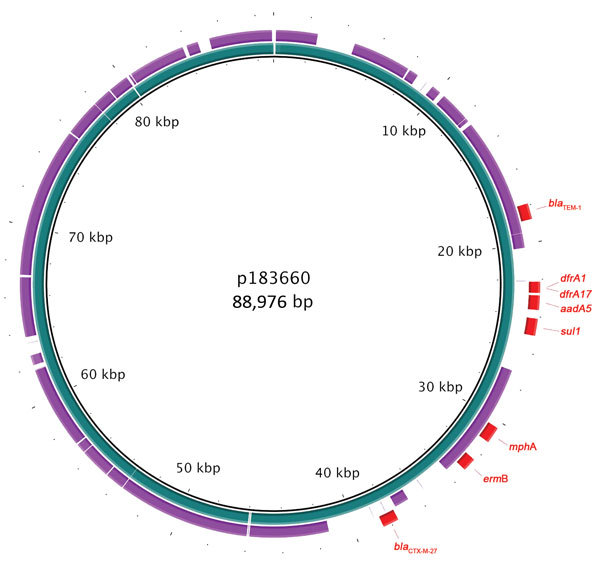
Genomic plot of multidrug-resistance plasmid p183660 (inner ring, blue) from a man in England infected with *Shigella sonnei* compared with pKSR100 (outer ring, purple), a multidrug-resistance plasmid from a case of *S. flexneri* 3a infection occurring among men who have sex in men (*4*). Drug-resistant elements from p183660 are shown in red. Plot produced by using BLAST Ring Image Generator (*5*).

DNA from isolate 183660 was used to elucidate plasmid sequencing (Technical Appendix). The multidrug-resistance phenotype was explained by the presence of an 89,000-bp IncFII plasmid designated p183660 (GenBank accession no. KX008967). This plasmid had >95% nt identity to pKSR100, a multidrug-resistance plasmid from an MSM case of *S. flexneri* 3a infection (GenBank accession no. LN624486) that had acquired the previously described pKSR100 integron (*bla*_TEM-1_, *dfrA17*, *dfrA1*, *sul1*, *aadA5*) (*4*) and a novel mobile element harboring *bla*_CTX-M-27_ (Figure 1). Macrolide resistance was conferred by *erm*B and *mph*A.

**Figure 2:**
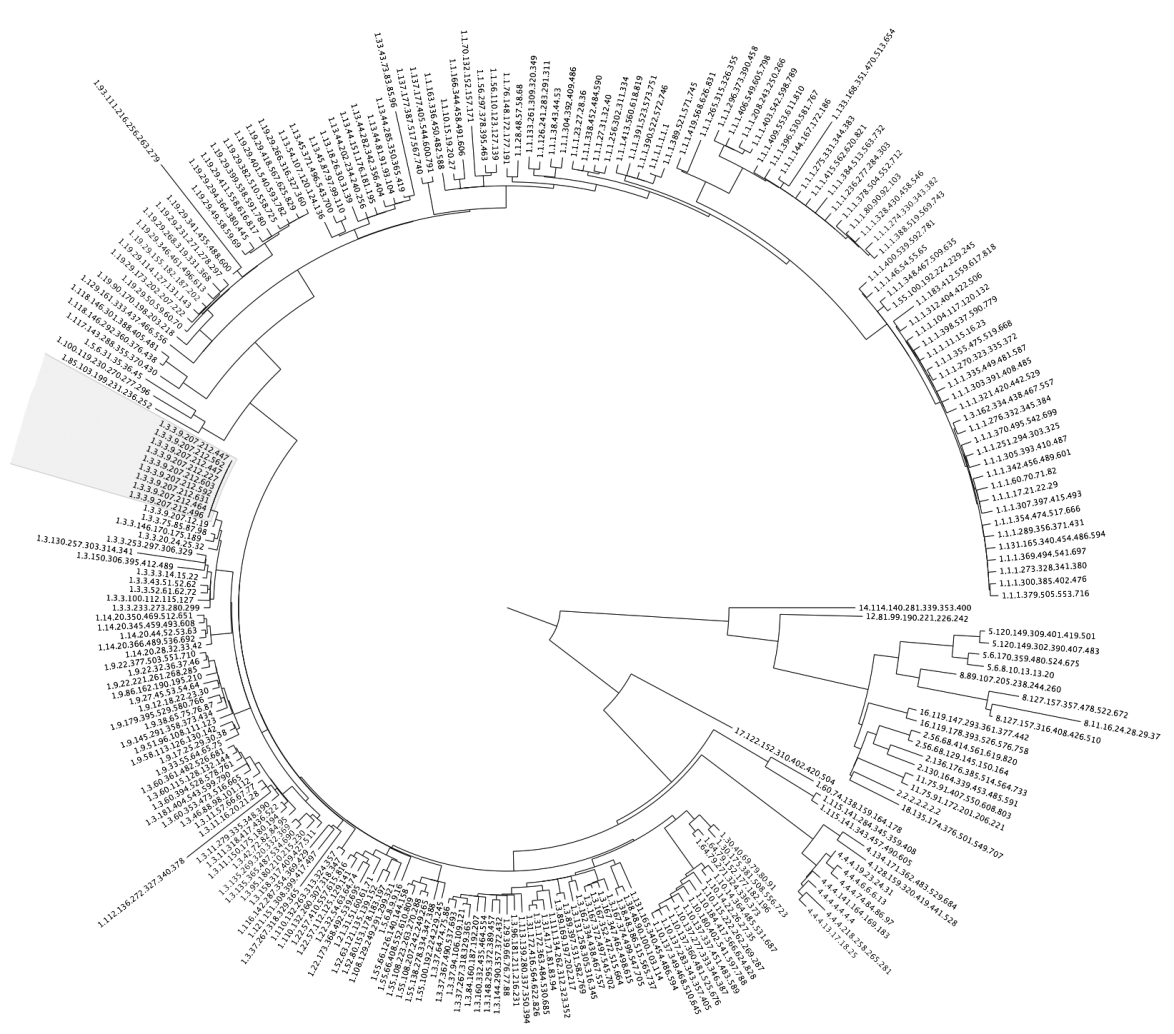
Maximum-likelihood phylogeny showing isolates from a cluster of 9 cases (gray shading) of *Shigella sonnei* infection among men who have sex with men in England, 2015. For context, 246 *S. sonnei* isolates that are representatives from each t25 cluster were included in the comparison. Isolates are labeled by single nucleotide polymorphism address.

For this investigation, we defined a case-patient as a resident of England with laboratory-confirmed *S. sonnei* infection belonging to a 5 SNP cluster with the SNP address 1.3.3.9.207.212.%, a resistance profile characterized by the presence of the plasmid p183660, and a specimen collected during September 1, 2015–February 29, 2016. We identified 9 case-patients (Figure 2) from fecal specimens; all had no known travel history in the 2 weeks before illness. Median age was 33 (range 28–83) years. Gastrointestinal symptom onset ranged from early September 2015 to late December 2015. No patients were bacteremic. Treatment failure was reported for 2 patients; both were hospitalized and 1 died , although *Shigella* was not recorded as a cause of death. Duration of illness was not systematically collected. Diagnoses occurred in general practice (n = 5), sexual health clinics (n = 2), and hospitals (n = 2). Seven patients resided in London; 2 resided elsewhere in England, 1 of whom reported recent travel to London. Of 8 patients with reported ethnicity, 6 self-identified as white.

In-depth questionnaires administered by telephone interviews collected information on sexual history, food exposures, and travel history; 8 patients completed the interviews. Seven answered detailed sexual history questions (including the 2 patients identified as MSM from clinical reporting data); all 7 reported having male partners. Six reported exposures considered to be high risk for *Shigella* transmission during the incubation period (e.g., fisting, sex under the influence of recreational drugs [*6*], oral-anal contact, attendance at sex parties, or use of sex toys with a partner) (Table 2). Reported number of sexual partners during the 2 weeks before illness was 0–5. The 7 patients who completed the sexual history questions reported previous high-risk sexual exposures; 6 of these patients and 1 patient who partially completed the questionnaire reported a history of other sexually transmitted infections, including gonorrhea, chlamydia, syphilis, nonspecific urethritis, hepatitis C, and pubic lice. Five reported using mobile apps to meet partners at some time in the past; none reported a prior shigellosis diagnosis. All had visited sexual health services (Table 2). Some patients were HIV positive (frequency is omitted to prevent deductive disclosure). 

**Table 2:** Reported exposure history for 9 case-patients with *Shigella sonnei* infection in the cluster with ESBL production and macrolide resistance, England, 2015*

Risk factor	No. (%) exposed	No. (%) unexposed		No. unknown†
Exposure ever before onset				
Self-identified as a man who has sex with men	7 (100)	0		2
Fisting	2 (29)	5 (71)		2
Sex under the influence of recreational drugs	4 (57)	3 (43)		2
Scat play	0	7 (100)		2
History of other sexually transmitted infections	7 (88)	1 (13)		1
Use of apps to meet partners	5 (71)	2 (29)		2
Awareness of *Shigella* infections	0	7 (100)		2
Previous *Shigella* diagnosis	0	8 (100)		1
Known to sexual health services	7 (100)	0		2
Exposure during the 2 weeks before onset				
Fisting	1 (14)		6 (86)	2
Sex under the influence of recreational drugs	3 (43)		4 (57)	2
Oral–anal contact	3 (43)		4 (57)	2
Condomless sex‡	5 (71)		2 (29)	2
Attended sex parties or live sex premises	3 (43)		4 (57)	2
Use of sex toys with partner	1 (14)		6 (86)	2
Scat play	0		7 (100)	2

*ESBL, extended-spectrum β-lactamase; scat play, sexual arousal or activity linked to feces.  †Unknowns were not included in percentage calculations; 7 of 9 patients completed and 1 partially completed interviews about sexual behavior. ‡Any condomless sexual contact.

No epidemiologic links were identified among patients, and no point sources were identified, although food exposure data was collected for 5 patients only. Two reported attending the same sex-on-premises venue in London 3 months apart (1 reported no sexual contact at the venue). None reported awareness of possible infection with *Shigella* spp. or of contact with others with gastrointestinal symptoms. Three thought they had acquired their infections from food. Patients reported that they accessed health promotion messages at sexual health clinics and on the internet.

## Conclusions

We describe a phylogenetic cluster of ESBL-producing *S. sonnei* infections in MSM in England that raises concerns about the ability to manage the spread of resistant *Shigella* infection in this population. Whole-genome sequencing revealed that the multidrug-resistance phenotype is conferred by acquisition of an IncFII plasmid (p183660) known to be circulating in *S*. *flexneri­–*affected MSM (*4*); this plasmid has acquired *bla*_CTX-M-27_.

Patient-reported behaviors were similar to those reported in an earlier *S. flexneri* epidemic among MSM: high numbers of sexual partners, high levels of condomless sex, attendance at sex parties, sex under the influence of recreational drugs, and prior HIV infection (*6*). We identified no point source, further indicating that sexual contact was the dominant mode of transmission.

Although *S. sonnei* causes self-limiting diarrhea in most patients, life-threatening invasive infections in patients co-infected with HIV have occurred (*7*). Quinolone and azithromycin resistance have been observed in recent outbreaks of *S. sonnei* infections in MSM; cepaholsporins are recognized as a suitable therapeutic choice for invasive or prolonged infections (*8*,*9*).

Primary public health concerns include possible treatment failure for severe shigellosis in immunocompromised patients; further spread of the multidrug-resistant IncFII plasmid to other enteric pathogens in MSM, with possible implications for treatment among the immunocompromised; rapid spread of drug-resistant *S. sonnei* infections among MSM, including outside the United Kingdom, as happened with *S. flexneri* 3a (*4*), especially among the HIV infected; and transmission through food handling and childcare centers, with potential to cause outbreaks of drug-resistant *S. sonnei* infections in other populations. Low awareness of *Shigella* infections among patients in our study suggests that prior awareness campaigns targeting high-risk MSM in England (*10*) have not been fully successful. Future campaigns being planned to improve awareness of *Shigella* infection and transmission routes will target social media, sexual health clinics, and primary care workers to increase awareness among MSM and healthcare staff. Frontline sexual health clinicians and microbiologists in England have been made aware of emerging drug resistance in *Shigella* spp. among this vulnerable group and of the need to perform antimicrobial drug susceptibility testing if treatment is considered necessary.

Technical Appendix Detailed methods for whole genome sequencing of *Shigella sonnei* isolates and plasmid elucidation.
