# Supplementary material for: ESBL-Producing and Macrolide-Resistant Shigella sonnei Infections among Men Who Have Sex with Men, England, 2015
Source: Emerg Infect Dis. 2016 Nov;22(11):1948–52. doi: 10.3201/eid2211.160653 (PMC5088027; doi:10.3201/eid2211.160653)
Supplement: Technical Appendix — Detailed methods for whole genome sequencing of Shigella sonnei isolates and plasmid elucidation. [file 16-0653-Techapp-s1.pdf]

Article DOI: http://dx.doi.org/10.3201/eid2211.160653

ESBL-Producing and Macrolide-Resistant *Shigella sonnei* Infections among Men Who Have Sex with Men, England, 2015

Technical Appendix

Detailed Methods

Public Health England conducted whole-genome sequencing of ≈70% of *S. sonnei* isolates voluntarily referred from hospital laboratories. In brief, DNA is extracted from cultures of *Shigella sonnei* isolates for sequencing on the HiSeq 2500 (Illumina, San Diego, USA). High-quality reads are mapped to the *S. sonnei* reference genome Ss046 (GenBank accession no. NC_007384.1) by using the Burrows-Wheeler Aligner's Smith-Waterman Alignment algorithm (*1*). Single nucleotide polymorphisms (SNPs) are identified by using the Genome Analysis Toolkit (*2*) in unified genotyper mode. Core genome positions with a high-quality SNP (>90% similarity, minimum depth [minimum number of reads needed to call a genotype] ×10, genotype quality >30) in at least 1 isolate are extracted, and maximum likelihood phylogeny is derived by using RaxML (*3*).

When necessary to elucidate plasmid sequence, DNA is extracted using the molecular Invisorb Spin Mini Kit (Stratec Biomedical) and is diluted to a concentration of 1 µg of genomic DNA in 50 µL of water. A MinION library (Oxford Nanopore Technologies, Oxford, UK) is prepared by using the SQK-MAP006 genomic sequencing kit (Nanopore), according to the manufacturer's instructions, and sequencing is performed on an Mk1 MinION device (Nanopore) with an Mk1 flow cell. A FASTA file of the readings is extracted with poretools (*4*) and SPAdes version 3.6.2 (*5*), which is used to produce a hybrid.

References

1. Li H, Durbin R. Fast and accurate long-read alignment with Burrows-Wheeler transform. Bioinformatics. 2010;26:589–95 http://dx.doi.org/10.1093/bioinformatics/btp698

2. McKenna A, Hanna M, Banks E, Sivachenko A, Cibulskis K, Kernytsky A, et al. The Genome Analysis Toolkit: a MapReduce framework for analyzing next-generation DNA sequencing data. Genome Res. 2010;20:1297–303 http://dx.doi.org/10.1101/gr.107524.110.

3. Stamatakis A. RAxML version 8: a tool for phylogenetic analysis and post-analysis of large phylogenies. Bioinformatics. 2014;30:1312–3 http://dx.doi.org/10.1093/bioinformatics/btu033.

4. Loman NJ, Quinlan AR. Poretools: a toolkit for analyzing nanopore sequence data. Bioinformatics. 2014;30:3399–401 http://dx.doi.org/10.1093/bioinformatics/btu555.

5. Bankevich A, Nurk S, Antipov D, Gurevich AA, Dvorkin M, Kulikov AS, et al. SPAdes: a new genome assembly algorithm and its applications to single-cell sequencing. J Comput Biol. 2012;19:455–77 http://dx.doi.org/10.1089/cmb.2012.0021.
